# Antidepressant-like effects of translocator protein (18 kDa) ligand ZBD-2 in mouse models of postpartum depression

**DOI:** 10.1186/s13041-018-0355-x

**Published:** 2018-03-05

**Authors:** Xu-bo Li, An Liu, Le Yang, Kun Zhang, Yu-mei Wu, Ming-gao Zhao, Shui-bing Liu

**Affiliations:** 1 Precision Pharmacy & Drug Development Center, Tangdu Hospital, Fourth Military Medical University, Xinsi Road 1, Xi’an, 710038 China; 2 Department of Pharmacology, School of Pharmacy, Fourth Military Medical University, Xi’an, 710032 China

**Keywords:** Translocator protein 18 kDa, ZBD-2, Postpartum depression, Amygdala

## Abstract

The 18 kDa translocator protein (TSPO) is primarily localized in the outer mitochondrial membrane of steroid-synthesizing cells in the central and peripheral nervous systems. One of the protein’s main functions is transporting substrate cholesterol into the mitochondria in a prerequisite process for steroid synthesis. Clinical trials have indicated that TSPO ligands might be valuable in treating some neuropathies and psychopathies. However, limited information is known about the role of TSPO in postpartum depression (PPD). The TSPO ligand ZBD-2, a derivative of XBD173, was synthesized in our laboratory. Behavioral tests, enzyme linked immunosorbent assay, and Western blot were employed to evaluate ZBD-2’s efficacy against PPD and to elucidate the potential underlying molecular mechanism. The TSPO levels significantly decreased in the basolateral amygdala of PPD models. After treatment for 2 weeks, ZBD-2 alleviated depression-like behaviors and enhanced the TSPO level in a PPD animal model. The underlying mechanisms of ZBD-2 were related to regulate the hypothalamic-pituitary-adrenal axis, enhance 5-HT and BDNF secretion, and maintain the excitatory and inhibitory synaptic protein expression to normal levels. Our results directly confirm that ZBD-2 exerts a therapeutic effect on PPD, which provides a new target for anti-PPD drug development.

## Introduction

Postpartum depression (PPD), a widespread mental disorder, occurs in women soon after giving birth [1]. Data have shown that approximately 40% of new mothers develop moderate to severe depression, and symptoms include sadness and hopelessness [2]. PPD is well known to negatively influence the offspring, which may then acquire deficits in cognitive and social interaction, as well as emotional disorders [3]. However, the underlying etiology remains largely unknown. Peptide and steroid hormones dramatically fluctuate during pregnancy and the postpartum period; these changes may exacerbate symptoms in vulnerable women. In women at risk for depression, many symptoms attack during the period encompassing large variations in estradiol and progesterone [4]*.* The estrogen and progesterone levels rise steeply in pregnant women and then decrease rapidly after birth. Moreover, immune system, hypothalamic-pituitary-adrenal (HPA) axis hormones, cytokines, and fatty acids are also involved in the occurrence and development of PPD [5]. To date, no effective therapeutic method is available to treat PPD. Mothers and their individual families face a dilemma between the side effects of PPD pharmacotherapy and the adverse effects of untreated depression on their offspring. Therefore, the ideal drugs treating postpartum depression with minimal side effects are required for both mothers and their infants.

Translocator protein (TSPO) is an 18 kDa protein, which is widely distributed in the outer membrane of mitochondria in central and peripheral tissue [6]. This protein was initially identified as a peripheral binding site for diazepam and later functionally and structurally distinguished from the central benzodiazepine receptor [7]. Numerous studies have shown that TSPO plays an important role in cholesterol transport and steroidogenesis. Meanwhile, steroid hormones modulate TSPO expression and activity in neurons [8]. At sites of injury, inflammation, and neuropathological conditions (stroke, Alzheimer’s disease, Parkinson’s disease, Huntington’s disease, multiple sclerosis, and amyotrophic lateral sclerosis), TSPO expression was robustly enhanced in reactive microglia and astrocytes. Therefore, TSPO ligands are commonly regarded as sensitive biomarkers of brain imaging for neuroinflammation [9]. TSPO ligands have anxiolytic and antidepressant effects without evident side effects of conventional benzodiazepines [10–12]. Our previous work showed that ZBD-2, a TSPO ligand, effectively relieves anxiety [13] and depression [14] in animal models. However, the role of ZBD-2 in PPD is limited.

The amygdala is involved in modulation of stress and emotional disorders. In the amygdala, the basolateral amygdala (BLA) is a critical component that receives most of the cortical and subcortical inputs. BLA contains two major types of glutamatergic principal neurons and GABAergic interneurons. In the present study, the effects of ZBD-2 on PPD were determined in the BLA of animal model. These positive results suggested that ZBD-2 reduced anxiety-like and depression-like behaviors may be through regulating the HPA axis, enhancing 5-HT secretion, and maintaining the excitatory and inhibitory synaptic protein expression to normal levels in the BLA.

## Results

### ZBD-2-mediated relief of anxiety- and depression-like behaviors in PPD models

**Fig. 1:**
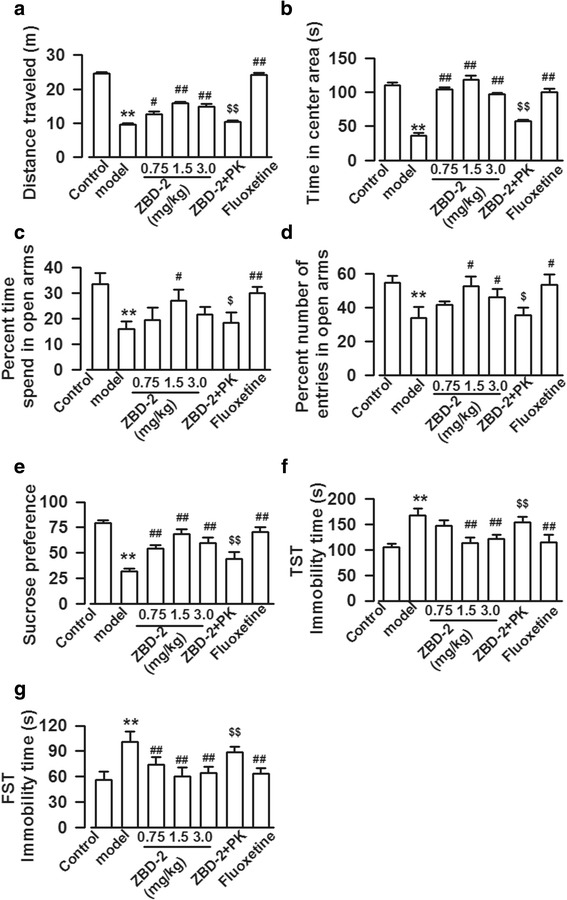
ZBD-2-mediated relief of anxiety- and depression-like behaviors in PPD models. **a**, **b** OF test showed that total distance traveled and the time in the central area were significantly reduced in the PPD mice as compared with those of the control. ZBD-2 relieved anxiety-like behaviors, as indicated by the increased total distance traveled and the time in the central area. PK11195 abolished the effects of ZBD-2 on total distance traveled and the time in the central area. **c**, **d** EPM test showed that the number of open-arm entries and the time spent in open arm were markedly decreased in the PPD models. ZBD-2 treatment reversed the number of open-arm entries and the time spent in open arm in the PPD mice. The effects of ZBD-2 were blocked by PK11195. **e-g** ZBD-2 obviously increased the sucrose intake in the PPD mice (**e**). In the FST and TST tests, ZBD-2 decreased immobility time in the PPD mice (**f**, **g**). The effects of ZBD-2 were abolished by PK11195 (**e-g**), and the effects of ZBD-2 (1.5 mg/kg) were comparable to those of fluoxetine in all behavior tests (**a-g**). *n* = 6 in each group. **p < 0.01 compared with the control; ^#^*p* < 0.05, ^##^*p* < 0.01 compared with PPD model; ^$^*p* < 0.05, ^$$^*p* < 0.01 compared with the ZBD-2 (1.5 mg/kg) group. PK: PK11195

In the OF test, the traveled distance and time in the central area were markedly reduced in the PPD model mice relative to those of the control mice (distance traveled: F_(6, 35)_ = 151.27, *P* < 0.001, LSD test; time in the central area: F_(6, 35)_ = 51.07, *P* < 0.001, Dunnett T3 test, Fig. 1a and b). Meanwhile, the PPD models showed decreased numbers of entries and time spent in open arms in the EPM test (percent time spent in open arms: F_(6,35)_ = 18.47, *P* < 0.001, LSD test; percent number of entries in open arms: F_(6,35)_ = 19.09, *P* < 0.001, LSD test, Fig. 1c and d). These data indicate that the PPD models exhibited anxiety-like behaviors. Next, we detected the depression-like behaviors through sucrose preference, TST, and FST tests. The sucrose consumption ratio was significantly decreased (F_(6,35)_ = 18.27, *P* < 0.001, LSD test, Fig. 1e), and the immobility times were markedly enhanced (TST immobility time: F_(6,35)_ = 26.22, *P* < 0.001, LSD test; FST immobility time: F_(6,35)_ = 19.61, *P* < 0.001, LSD test, Fig. 1f and g) in the PPD animal models than those of the control mice. These results suggest that the PPD animal models suffered from co-morbid depression and anxiety. ZBD-2 significantly relieved anxiety-like behaviors, as shown by the increased distance traveled and time in the central area during the OF test (Fig. 1a, b), as well as the increased open-arm entries and time spent in open arms in the EPM test (Fig. 1c, d). ZBD-2 treatment also ameliorated depression-like behaviors, as shown by the increased sucrose intake and reduced immobility times (Fig. 1e, f and g). The effects of ZBD-2 (1.5 mg/kg) were comparable with those of fluoxetine (a commonly used antidepressant in clinical) (*P*>0.05, Fig. 1b-g). The effects of ZBD-2 were blocked by PK11195 (a selective antagonist of TSPO), which indicated that ZBD-2 takes effect through activating TSPO.

### Effects of ZBD-2 on HPA axis hormones in PPD models

**Fig. 2:**
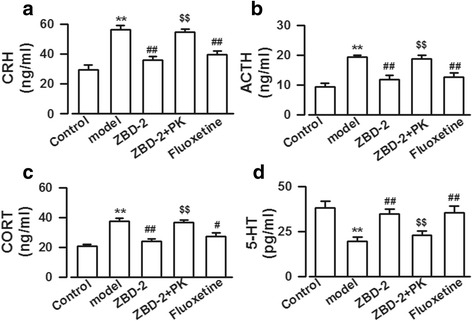
ZBD-2-mediated regulation of the HPA axis hormone levels in the PPD models. **a-d** The CRH (**a**), ACTH (**b**), and CORT (**c**) levels were significantly increased and the 5-HT (**d**) level was obviously decreased in the sera of PPD models. ZBD-2 reversed the CRH, ACTH, CORT, and 5-HT to basal levels, which were blocked by PK11195 treatment. The effects of ZBD-2 were comparable to the effects of fluoxetine. *n* = 6 mice in each group. **p < 0.01 compared with the control; ^#^*p* < 0.05, ^##^*p* < 0.01 compared with the PPD model; ^$$^*p* < 0.01 compared with the ZBD-2 group. PK: PK11195

We detected the levels of HPA axis hormones because they are involved in the occurrence and development of PPD [15]. The dose (1.5 mg/kg) of ZBD-2 was employed in the following experiments on the basis of above behavioral tests. The levels of CRH, ACTH, and CORT were obviously increased (CRH: F_(4,25)_ = 40.58, *P* < 0.001, LSD test; ACTH: F_(4,25)_ = 38.28, *P* < 0.001, LSD test; CORT: F_(4,25)_ = 17.41, *P* < 0.001, LSD test, Fig. 2a, b and c), and level of 5-HT was markedly decreased in the sera of PPD models as compared to those of control mice (F_(4,25)_ = 7.35, *P* < 0.001, LSD test, Fig. 2d). ZBD-2 significantly reverted the CRH, ACTH, CORT, and 5-HT concentrations to basal levels (Fig. 2). However, PK11195 treatment abolished the effects of ZBD-2 (*P* < 0.001, Fig. 2). There was no difference between the effects of fluoxetine and ZBD-2 (*P*>0.05, Fig. 2).

### Effects of ZBD-2 on TSPO, 5-HT receptors, and the neural plasticity protein BDNF in PPD mice

**Fig. 3:**
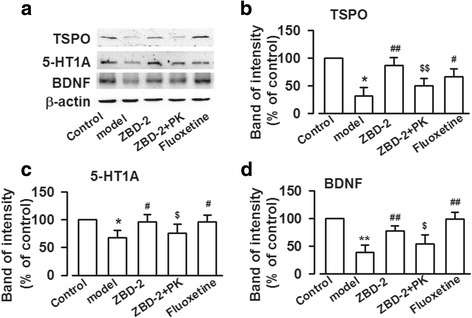
ZBD-2 increased the TSPO, 5-HT1A, and BDNF levels in PPD mice. **a** The representative Western blot analysises for TSPO, 5-HT1A, and BDNF were shown in the BLA. **b**, **c**, **d** ZBD-2 increased the levels of TSPO, 5-HT1A, and BDNF in the BLA of PPD mice. Similarly, PK11195 abolished the effects of ZBD-2, and the effects of ZBD-2 were comparable to those of fluoxetine. *n* = 5 in each group. *p < 0.05, **p < 0.01 compared with the control; ^#^*p* < 0.05, ^##^*p* < 0.01 compared with the PPD model; ^$^*p* < 0.05, ^$$^*p* < 0.01 compared with the ZBD-2 group. PK: PK11195

To clarify the molecular mechanism of ZBD-2 on PPD, the levels of TSPO, 5-HT receptor subtype 1A (5-HT1A) and BDNF were measured by Western blot. The results showed that the levels of TSPO, 5-HT1A, and BDNF were significantly reduced in the BLA of PPD models. (TSPO: F_(4,25)_ = 25.52, *P* < 0.001, LSD test; 5-HT1A: F_(4,25)_ = 8.90, *P* < 0.001, LSD test; BDNF: F_(4,25)_ = 56.47, *P* < 0.001, LSD test, Fig. 3a, b, c and d). Treatment with ZBD-2 (1.5 mg/kg) upregulated the levels of TSPO, 5-HT1A and BDNF in PPD models (Fig. 3a, b, c and d). The effects of ZBD-2 were comparable with the effects of fluoxetine (*P*>0.05, Fig. 3), and the effects of ZBD-2 on TSPO, 5-HT1A, and BDNF were blocked by PK11195 (*P* < 0.001, Fig. 3b; *P* < 0.05, Fig. 3c, d).

### Effects of ZBD-2 on the excitatory glutamic receptors in the BLA of PPD mice

**Fig. 4:**
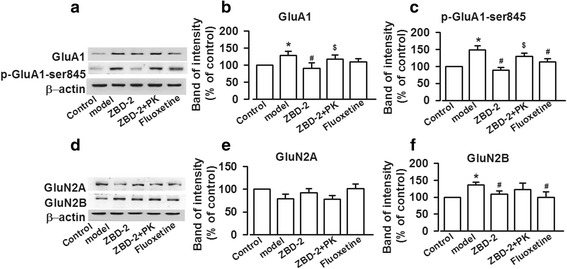
ZBD-2-induced reversal of glutamate receptor expression in PPD mice. **a**, **d** The representative Western blot analysises for GluA1, *p*-GluA1-Ser845, GluN2A, and GluN2B are presented in the BLA. **b**, **c**, **f** ZBD-2 administration reversed the increased levels of GluA1 (**b**), *p*-GluA1-Ser845 (**c**), and GluN2B (**f**) expression in the PPD model mice. The effects of ZBD-2 were blocked by PK11195. **e** ZBD-2 did not affect the levels of GluN2A-containing NMDA receptors in the BLA of PPD mice. There were no differences between ZBD-2 and fluoxetine treatment groups. *n* = 5 in each group. *p < 0.05 compared with the control; ^#^*p* < 0.05 compared with the PPD model; ^$^*p* < 0.05 compared with the ZBD-2 group. PK: PK11195

PPD is closely related to neurotransmitter disorder in CNS [5]. AMPA and NMDA receptors are two major glutamate receptors in CNS [16]. Therefore, the levels of AMPA and NMDA receptors were detected in the BLA. The levels of GluA1, phosphorylation of GluA1 at ser845 site (*p*-GluA1-Ser845), and GluN2B were enhanced in the BLA of PPD models (GluA1: F_(4,25)_ = 3.285, *P* = 0.072, LSD test; *p*-GluA1-Ser845: F_(4,25)_ = 3.687, *P* = 0.061, Dunnett T3 test; GluN2B: F_(4,25)_ = 4.431, *P* = 0.077, LSD test, Fig. 4b, c, and f), which were reversed by ZBD-2 administration (Fig. 4b, c, and f). In addition, treatment with PK11195 abolished the effects of ZBD-2 (*P* < 0.05, Fig. 4b, c). However, the level of GluN2A was not influenced in the BLA of PPD model with or without ZBD-2 and PK11195 treatment (Fig. 4e). The effects of ZBD-2 were comparable with the effects of fluoxetine (*P*>0.05, Fig. 4).

### Effects of ZBD-2 on GABA receptors in PPD mice

**Fig. 5:**
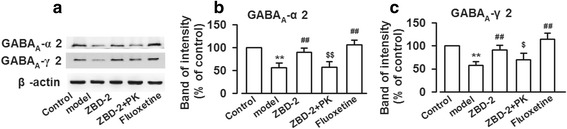
ZBD-2-induced reversal of the decreased expression of GABAA receptors in the PPD mice. **a** The representative Western blot analysises of GABAergic proteins are displayed in the BLA. **b**, **c** GABA_A_-α2 and GABA_A_-γ2 levels were decreased in the BLA of PPD mice. ZBD-2 administration reversed the downregulation of GABAA-α2 and GABAA-γ2 expression in the PPD mice. The effects of ZBD-2 were abolished by PK11195. There were no differences between ZBD-2 and fluoxetine treatment groups. *n* = 5 in each group. *p < 0.05, **p < 0.01 compared with the control; ^#^*p* < 0.05, ^##^*p* < 0.01 compared with the PPD model; ^$^*p* < 0.05, ^$$^*p* < 0.01 compared with the ZBD-2 (1.5 mg/kg) group. PK: PK11195

GABA is an important inhibitory neurotransmitter in CNS, and imbalance of excitatory and inhibitory transmission contributes to mental disorder [17]. PPD decreased the levels of GABA_A_-α2 and GABA_A_-γ2 in the BLA (GABA_A_-α2: F_(4,25)_ = 21.016, *P* = 0.002, Dunnett T3 test; GABA_A_-γ2: F_(4,25)_ = 3.821, *P* = 0.015, LSD test, Fig. 5a, b, and c), whereas ZBD-2 treatment upregulated their levels (Fig. 5a, b, and c). The effects of ZBD-2 were similar with those of fluoxetine (*P*>0.05), and PK11195 administration abolished the effects of ZBD-2 on GABA_A_-α2 and GABA_A_-γ2 levels (*P* < 0.001, Fig. 5b; *P* < 0.05, Fig. 5c).

## Discussion

In the present study, ZBD-2 treatment significantly ameliorated anxiety- and depression-like behaviors in the PPD mice. The underlying mechanisms of ZBD-2 included regulating HPA axis hormones, the levels of 5-HT, BDNF, AMPA, NMDA, and GABA_A_ receptors in the BLA of PPD animals.

### ZBD-2-mediated relief of anxiety- and depression-like behaviors in PPD models

PPD is the major mood disorder in the postpartum period; up to 50% of women experiences various degrees of PPD [18]. However, the pathogenesis of PPD is highly complex and has not been completely clarified. In present study, a PPD model was established by hormone-simulated pregnancy (HSP) to evaluate the effects of ZBD-2 on PPD. The “ovarian steroid withdrawal” hypothesis is based on the fact that significant variation in the levels of estrogen, progesterone, monoamine neurotransmitters and receptors, metabolic products, and other biological factors within the HPA axis cause the occurrence of PPD [15]. HSP-treated rats mimicked the high estrogen and progesterone levels in late pregnancy that rapidly declined after delivery. The decline in circulating ovarian steroids caused postpartum symptoms [19, 20]. In this study, PPD mice exhibited anxiety- and depression-like behaviors in EPM, OF, FST, and TST tests, indicating that HSP was suitable to establish PPD animal model.

The anxiolytic and antidepressant effects of ZBD-2 in the PPD model mice were blocked by PK11195, a potent TSPO antagonist, suggesting the effects of ZBD-2 are through activating TSPO. Previous studies have shown that TSPO ligands can increase the concentrations of pregnenolone, progesterone, and 3α,5α-tetrahydroprogesterone (3α, 5α-THP) in brain. XBD173, a TSPO ligand, has been shown to potentiate GABA-mediated neurotransmission and is a promising therapeutic drug for mental disorder [8]. Fluoxetine, a first-line antidepressant drug, was used as positive control in present study. It is a selective 5-HT reuptake inhibitor and is a modulator of neurosteroidogenesis in brain [21]. It has been found that fluoxetine also alleviates many premenstrual dysphoric disorder symptoms, which is strongly associated with ovarian hormones including progesterone [22]. Fluoxetine increases the 3α,5α-THP levels in the brains of depressed patients [23], and 3α,5α-THP is a potent positive allosteric modulator of the GABA_A_ receptor [24]. Accordingly, fluoxetine exerts its antidepressant effect partially through elevating neurosteroid levels and regulating GABAergic neurotransmission in brain.

### Mechanism of ZBD-2 on PPD

The HPA axis is essential in restoring normal homeostatic function following psychological stress [25]. It also plays a key role in the postpartum period [26, 27]. The activity of HPA axis is altered during pregnancy and postpartum stress [28]. HPA axis hormones are mainly composed of CRH, ACTH, and CORT, which are dysregulated in PPD patients [29]. The declining levels of monoamines, especially 5-HT, lead to functional and structural neuronal weakness, which cannot adapt to the stimulation of stress. Meanwhile, cognitive function and emotional regulation disorders and is accompanied by neuroendocrine immune dysfunction, eventually leading to depression [29]. ZBD-2 significantly recovered CRH, ACTH, CORT, and 5-HT concentrations to basal levels in the PPD models; thus, ZBD-2 is involved in regulating the HPA axis hormones and 5-HT.

TSPO is involved in neurodegenerative diseases and psychiatric disorders [30]. Moreover, TSPO level is enhanced in multiple sclerosis, amyotrophic lateral sclerosis, Parkinson’s disease, Huntington’s disease, AD, and stroke [31]. However, reduced TSPO expression has been observed in patients with co-morbidities of anxiety and depression or bipolar disorder [32] and has been associated with distress and aggression [33]. Our data showed that the TSPO levels were downregulated in the BLA of PPD models and restored to basal levels after ZBD-2 treatment. Therefore, TSPO downregulation is a possible response to PPD. We also found that the effect of ZBD-2 on PPD was comparable to that of fluoxetine and suggested the high complexity of PPD pathogenesis. Thus, multiple target therapy is needed beside 5-HT reuptake inhibitors.

BDNF, a neurotrophin related to the modulation of synaptic plasticity and long-term potentiation in brain [34], is essential to placental development during pregnancy and involved in major depression [35]. BDNF level decrease induced by stress results in aberrant neurogenesis and subsequent depression [36, 37], whereas its increase follows anti- depressant treatment [38]. BDNF is also reduced during pregnancy and in the postpartum period when concomitant with depressive symptoms [39]. Diminished BDNF levels are believed to be a potential pathological mechanism underlying the impaired neurogenesis in depression [40]. We found that administering ZBD-2 reversed the downregulation of BDNF expression in the BLA of PPD models. This result suggested that the antidepressant effect of ZBD-2 is closely related to the correction of abnormal BDNF levels in the BLA.

### ZBD-2-mediated amelioration of the imbalance in GABAergic and glutamatergic transmission

The balance between excitatory and inhibitory neurotransmitters is the basis of normal neurological CNS function. Glutamate is a major excitatory neurotransmitter and GABA is the most important inhibitory neurotransmitter in brain [41]. The large increase in progesterone-derived neurosteroids during pregnancy and their precipitous decrease at parturition may have considerable effects on GABA_A_Rs during pregnancy and postpartum [42]. Glutamate levels are sensitive to ovarian hormone fluctuations, pregnancy and the postpartum period [43]. The dysfunction of GABAergic and glutamatergic transmission is connected with PPD [41]. In the BLA of PPD mice, the levels of excitatory glutamate receptors including GluA1, *P*-GluA1-Ser845, and GluN2B increased, whereas those of inhibitory GABA_A_-α2 and GABA_A_-γ2 receptor decreased. Meanwhile, ZBD-2 significantly reversed these alterations but did not affect the levels of GluN2A- containing NMDA receptors in the BLA of PPD mice. The difference between GluN2A and GluN2B levels suggests that GluN2A and GluN2B play different roles in the development of PPD [44]. Synaptic and extrasynaptic NMDA receptors couple different intracellular signaling pathways [45]. Thus, the effects of ZBD-2 against PPD may be due to modulate the balance between excitatory and inhibitory transmission in the BLA.

In conclusion, we investigated the effects and potential molecular mechanisms of ZBD-2 on PPD treatment. The mechanisms of ZBD-2 are related to regulate the HPA axis, enhance 5-HT and BDNF secretion, and maintain the excitatory and inhibitory synaptic protein expressions to normal levels. These results provide important evidence that TSPO level in the BLA is involved in PPD development and ZBD-2 is an effective antidepressant drug against PPD.

## Methods

### Materials

ZBD-2 was prepared at our laboratory as previously described [13]. Anti-*β*-actin antibody was purchased from Sigma (St. Louis, MO). Anti-GluN2A, anti-GluN2B, anti-GluA1, anti-*p*-GluA1-ser845, anti-5-HT1A and anti-BDNF antibodies were purchased from Abcam (Cambridge, UK). Anti-TSPO, anti-GABA_A_-α2 and anti-GABA_A_-γ2 antibodies were purchased from Chemicon (Temecula, CA, USA). All secondary antibodies conjugated with horseradish peroxidase (HRP) were purchased from Santa Cruz Biotechnology (Santa Cruz, CA, USA). The CRH (Corticotropin-releasing hormone), ACTH (Adreno-cortico- tropic-hormone), CORT (Corticosterone) and 5-HT (5-hydroxytryptamine) ELISA kits were purchased from (Cusabio, Wuhan, China). All of the chemicals and reagents used were standard biochemical quality and commercially available.

### Animals

Adult (8-week old) female C57BL/6 mice, weighing 18–22 g, were used in this experiment. The animals were housed in groups with rodent diet and water ad libitum. The holding room was maintained at room temperature at 22–25 °C with humidity (50%–60%) and 12-h light/day cycle. All experimental procedures were approved by the Animal Ethics Committee of the Fourth Military Medical University.

### Surgical procedures

At beginning of the experiment, adult mice were bilaterally ovariectomized (OVX). The surgery was performed using aseptic techniques while under 4% chloral anesthesia. One dorsal lateral incision of waist was made for each side, the ovaries were isolated and sterile suture was tied tightly around the ovaries, and the ovaries were removed. The muscle layer and the cutaneous incision were sutured separately [46]. The mice were allowed to recover for at least 7 days prior to next procedure.

### Hormone-simulated pregnancy

**Fig. 6:**
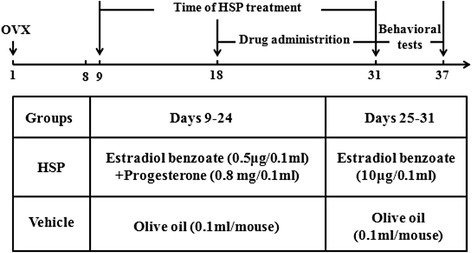
Timeline of the experiment and HSP was employed to establish PPD mouse model

After one-week recovery following the OVX, mice were administered hormones (estradiol and progesterone dissolved in 0.1 ml sesame oil) for 23 days to establish hormone-simulated pregnancy (HSP) as shown in Fig. 6 [46]. The “ovarian steroid withdrawal” hypothesis is based on the onset of PPD when the levels of estrogen and progesterone are rapidly decreased after delivery. The sham group was subcutaneously injected with the same volume sesame oil (vehicle) after OVX.

### Drug treatment

The animals received administration of vehicle or ZBD-2 (0.75 mg/kg, 1.5 mg/kg, 3.0 mg/ kg, p.o.), PK11195 (3.0 mg/kg, i.p.) and Fluoxetine (3.0 mg/kg, p.o.) once daily for 2 weeks (from Day 18 to Day 31). Behavioral tests were performed 1 h after the last administration. The samples of BLA were dissected immediately after behavioral tests.

### Behavioral tests

Before the behavioral tests, mice were pretreated with mild stroking two times per day for 7 consecutive days to eliminate their nervousness. On the day of behavioral tests, the mice were moved to behavioral testing room at least 1 h for habituation.

#### Open field test (OFT)

The OFT was evaluated by analyzing the spontaneous activity of mice in open space [47]. Mice were gently placed in the center of an open acrylic box (30 cm × 30 cm × 30 cm) with clear plexiglas walls and white floor, and allowed mice to freely explore for 10 min. The trial for each subject was recorded in a 15 min session by a camera and the data were analyzed with a video-tracking system. The central square area of the box (one fourth of the whole area) was defined as the center zone.

#### Elevated plus maze (EPM)

The EPM apparatus is consisted of two open arms (without walls) and two enclosed arms (with walls). Each arm was 25 cm long and 8 cm wide, and the wall for the closed arm was 12 cm high. The maze is raised to a height of 50 cm above the ground. The mice were placed in the central square with their heads facing open arms and allowed mice to explore freely for 5 min. The numbers of arm entries and total time spent in open and enclosed arms were recorded by video for 5 min, and analyzed by video tracking system [14].

#### Sucrose preference test (SPT)

SPT was performed as previously described [48]. All mice were housed individually in a cage and deprived of water for 18 h, and then two bottles were placed in each cage (one is water and the other is 1% sucrose solution). The animal were allowed to drink ad libitum for 24 h [49]. To avoid position preference, two bottles were interchanged every 6 h according to previous report [50]. The consumption of water and sucrose solution was recorded during the 24 h, and the sucrose preference of each mouse was calculated as: sucrose preference (%) = sucrose solution intake/total liquid consumption× 100%.

#### Tail suspension test (TST)

TST was conducted as previously described [51]. The mouse was individually suspended 15 cm above the floor by the tail with adhesive tape placed approximately 2 cm from tail tip. The short-term inescapable stress led to an immobile posture. The animal behavior was recorded by video for 6 min. The performance was analyzed during the last 5 min.

#### Forced swim test (FST)

The apparatus of FST is a glass cylinder (30 cm diameter × 66 cm height) and filled with water for 25 cm depth at 25 ± 1 °C in a quiet testing room. On FST day 1, animals were individually placed into the water of glass cylinder for 15 min to induce a state of helplessness. 24 h later, the mice were returned to the same condition for 6 min, and three types of behaviors were observed in the last 5 min: immobility, swimming, and struggling. After each FST trial, the mouse was towel-dried, kept warm, and then returned to its home cage [52]. Water was changed after each animal test to avoid any influence.

### Enzyme linked immunosorbent assay (ELISA)

The levels of CRH, ACTH, CORT, and 5-HT in serum were determined with commercially available ELISA kit according to the manufacturer instructions. Briefly, the mice were anesthetized with diethyl ether, and then used ophthalmological forceps to remove the eyeballs. Blood (about 1.0 ml/mouse) was collected in the 1.5 ml centrifuge tubes (anticoagulation with heparin). Then tubes were centrifuged at 3000 rpm at 4 °C for 10 min to isolate serum, and the supernatant was collected for ELISA. Serum was stored at − 80 °C until use.

### Western blot

The expressions of proteins in the BLA were analyzed using Western blotting [13]. The BLA were quickly dissected from brain slices (300 mm) on an ice-cold plate under an anatomical microscope, then the BLA was immediately placed in the labeled 1.5 ml centrifuge tube, weighed, and homogenized in cold tissue lysate (containing 1% 10 mM PMSF). The homogenates were centrifuged at 12,000 rpm with 4 °C for 15 min, and 5 × loading buffer was added into supernatants and heated at 95 °C for 5 min. The samples were stored at − 80 °C until use. After centrifugation, 50 μg protein were separated and electrotransferred onto PDVF membranes (Invitrogen), which were probed with antibody for TSPO (1:2000 dilution), anti-5-HT1A (1:500), GluN2A (1:500), GluN2B (1:500), GluA1 (1:300), P-GluA1-ser845 (1:1000), GABA_A_-γ (1:500), GABA_A_-α (1:500), and BDNF (1:500) with β-actin (1:10000) as the loading control. The membranes were incubated with secondary antibodies anti-rabbit/anti-mouse/anti-goat IgG for the primary antibodies). The quantity of band intensity was normalized by comparison with β-actin, and the density analysis of protein was conducted using an ECL system (Lightning Blot System, PerkinElmer, Waltham, MA, USA).

### Statistical analysis

Experimental Data were analyzed using SPSS 13.0. Results were expressed as the mean ± SEM. Data passed the homogeneity test were analyzed by the one-way ANOVA least significant difference (LSD) test, otherwise were analyzed by the one-way ANOVA Dunnett’s T3 test comparisons. *P* < 0.05 was considered statistically significant.

## Abbreviations

5-HT: 5-hydroxytryptamine
ACTH: Adreno-cortico-tropic-hormone
Adr: Adrenaline
BDNF: Brain-derived neurotrophic factor
BLA: Basolateral amygdala
CNS: Central nervous system
CORT: Corticosterone
CREB: cAMP response element binding protein
CRH: Corticotropin-releasing hormone
E: Estrogen
EPM: Elevated plus maze
ER: Estrogen receptor
FST: Forced swimming test
GABA: γ-aminobutyric acid
Gln: Glutamine
Glu: Glutamate
HPA: Hypothalamic- pituitary-adrenal
HPA: Hypothalamo-pituitary-adrenal
HPG: Hypothalamic-pituitary-gonadal
HPT: Hypothalamic- pituitary-thyroid
HSP: Hormone-simulated pregnancy
LTP: Long-term potentiation
NE: Norepinephrine
NMDA: N-Methyl-D-aspartic acid
OF: Open field test
OVX: Ovariectomy
P: Progesterone
PPD: Postpartum depression
SPT: Sucrose preference test
TSPO: Translocator protein 18 kDa
TST: Tail suspension test
WB: Western blot

## References

[1] Husain N, Bevc I, Husain M, Chaudhry IB, Atif N, Rahman A (2006). Prevalence and social correlates of postnatal depression in a low income country. Arch Womens Ment Health.

[2] Hucke EE, Cruz-Casallas PE, Sider LH, Felicio LF (2001). Reproductive experience modulates dopamine-related behavioral responses. Pharmacol Biochem Behav.

[3] Iles J, Slade P, Spiby H (2011). Posttraumatic stress symptoms and postpartum depression in couples after childbirth: the role of partner support and attachment. J Anxiety Disord.

[4] Moses-Kolko EL, Berga SL, Kalro B, Sit DK, Wisner KL (2009). Transdermal estradiol for postpartum depression: a promising treatment option. Clin Obstet Gynecol.

[5] Zonana J, Gorman JM (2005). The neurobiology of postpartum depression. CNS Spectr.

[6] Culty M, Li H, Boujrad N, Amri H, Vidic B, Bernassau JM, Reversat JL, Papadopoulos V (1999). In vitro studies on the role of the peripheral-type benzodiazepine receptor in steroidogenesis. J Steroid Biochem Mol Biol.

[7] Choi J, Ifuku M, Noda M, Guilarte TR (2011). Translocator protein (18 kDa)/peripheral benzodiazepine receptor specific ligands induce microglia functions consistent with an activated state. Glia.

[8] Rupprecht R, Papadopoulos V, Rammes G, Baghai TC, Fan J, Akula N, Groyer G, Adams D, Schumacher M (2010). Translocator protein (18 kDa) (TSPO) as a therapeutic target for neurological and psychiatric disorders. Nat Rev Drug Discov.

[9] Murail S, Robert JC, Coic YM, Neumann JM, Ostuni MA, Yao ZX, Papadopoulos V, Jamin N, Lacapere JJ (2008). Secondary and tertiary structures of the transmembrane domains of the translocator protein TSPO determined by NMR. Stabilization of the TSPO tertiary fold upon ligand binding. Biochim Biophys Acta.

[10] Rupprecht R, Rammes G, Eser D, Baghai TC, Schule C, Nothdurfter C, Troxler T, Gentsch C, Kalkman HO, Chaperon F (2009). Translocator protein (18 kD) as target for anxiolytics without benzodiazepine-like side effects. Science.

[11] Zhang LM, Zhao N, Guo WZ, Jin ZL, Qiu ZK, Chen HX, Xue R, Zhang YZ, Yang RF, Li YF (2014). Antidepressant-like and anxiolytic-like effects of YL-IPA08, a potent ligand for the translocator protein (18 kDa). Neuropharmacology.

[12] Costa B, Da Pozzo E, Martini C (2012). Translocator protein as a promising target for novel anxiolytics. Curr Top Med Chem.

[13] Wang DS, Tian Z, Guo YY, Guo HL, Kang WB, Li S, Den YT, Li XB, Feng B, Feng D (2015). Anxiolytic-like effects of translocator protein (TSPO) ligand ZBD-2 in an animal model of chronic pain. Mol Pain.

[14] Wang DS, Han J, Li S, Sun T, Guo YY, Kang WB, Tian Z, Zhao JN, Liu G, Liu SB, Zhao MG (2017). Antidepressant-like and anxiolytic-like effects of ZBD-2, a novel ligand for the translocator protein (18 kDa). NeuroMolecular Med.

[15] Ferguson EH, Di Florio A, Pearson B, Putnam KT, Girdler S, Rubinow DR, Meltzer-Brody S (2017). HPA axis reactivity to pharmacologic and psychological stressors in euthymic women with histories of postpartum versus major depression. Arch Womens Ment Health.

[16] Bhandage AK, Jin Z, Hellgren C, Korol SV, Nowak K, Williamsson L, Sundstrom-Poromaa I, Birnir B (2017). AMPA, NMDA and kainate glutamate receptor subunits are expressed in human peripheral blood mononuclear cells (PBMCs) where the expression of GluK4 is altered by pregnancy and GluN2D by depression in pregnant women. J Neuroimmunol.

[17] Sartorius A, Mahlstedt MM, Vollmayr B, Henn FA, Ende G (2007). Elevated spectroscopic glutamate/gamma-amino butyric acid in rats bred for learned helplessness. Neuroreport.

[18] Miller LJ (2002). Postpartum depression. JAMA.

[19] Stoffel EC, Craft RM (2004). Ovarian hormone withdrawal-induced “depression” in female rats. Physiol Behav.

[20] Furuta M, Numakawa T, Chiba S, Ninomiya M, Kajiyama Y, Adachi N, Akema T, Kunugi H (2013). Estrogen, predominantly via estrogen receptor alpha, attenuates postpartum-induced anxiety- and depression-like behaviors in female rats. Endocrinology.

[21] Pinna G, Costa E, Guidotti A (2006). Fluoxetine and norfluoxetine stereospecifically and selectively increase brain neurosteroid content at doses that are inactive on 5-HT reuptake. Psychopharmacology.

[22] Su TP, Schmidt PJ, Danaceau MA, Tobin MB, Rosenstein DL, Murphy DL, Rubinow DR (1997). Fluoxetine in the treatment of premenstrual dysphoria. Neuropsychopharmacology.

[23] Romeo E, Strohle A, Spalletta G, di Michele F, Hermann B, Holsboer F, Pasini A, Rupprecht R (1998). Effects of antidepressant treatment on neuroactive steroids in major depression. Am J Psychiatry.

[24] Lambert JJ, Belelli D, Peden DR, Vardy AW, Peters JA (2003). Neurosteroid modulation of GABAA receptors. Prog Neurobiol.

[25] Gourley SL, Taylor JR (2009). Recapitulation and reversal of a persistent depression-like syndrome in rodents. Curr Protoc Neurosci.

[26] Glynn LM, Davis EP, Sandman CA (2013). New insights into the role of perinatal HPA-axis dysregulation in postpartum depression. Neuropeptides.

[27] Duthie L, Reynolds RM (2013). Changes in the maternal hypothalamic-pituitary-adrenal axis in pregnancy and postpartum: influences on maternal and fetal outcomes. Neuroendocrinology.

[28] Meinlschmidt G, Martin C, Neumann ID, Heinrichs M (2010). Maternal cortisol in late pregnancy and hypothalamic-pituitary-adrenal reactivity to psychosocial stress postpartum in women. Stress.

[29] Jolley SN, Elmore S, Barnard KE, Carr DB (2007). Dysregulation of the hypothalamic- pituitary- adrenal axis in postpartum depression. Biol Res Nurs.

[30] Arbo BD, Benetti F, Garcia-Segura LM, Ribeiro MF (2015). Therapeutic actions of translocator protein (18 kDa) ligands in experimental models of psychiatric disorders and neurodegenerative diseases. J Steroid Biochem Mol Biol.

[31] Chen MK, Guilarte TR (2008). Translocator protein 18 kDa (TSPO): molecular sensor of brain injury and repair. Pharmacol Ther.

[32] Abelli M, Chelli B, Costa B, Lari L, Cardini A, Gesi C, Muti M, Lucacchini A, Martini C, Cassano GB, Pini S (2010). Reductions in platelet 18-kDa translocator protein density are associated with adult separation anxiety in patients with bipolar disorder. Neuropsychobiology.

[33] Ritsner M, Modai I, Gibel A, Leschiner S, Silver H, Tsinovoy G, Weizman A, Gavish M (2003). Decreased platelet peripheral-type benzodiazepine receptors in persistently violent schizophrenia patients. J Psychiatr Res.

[34] Nagahara AH, Tuszynski MH (2011). Potential therapeutic uses of BDNF in neurological and psychiatric disorders. Nat Rev Drug Discov.

[35] Christian LM, Mitchell AM, Gillespie SL, Palettas M (2016). Serum brain-derived neurotrophic factor (BDNF) across pregnancy and postpartum: associations with race, depressive symptoms, and low birth weight. Psychoneuroendocrinology.

[36] Duman RS, Monteggia LM (2006). A neurotrophic model for stress-related mood disorders. Biol Psychiatry.

[37] Duman RS, Heninger GR, Nestler EJ (1997). A molecular and cellular theory of depression. Arch Gen Psychiatry.

[38] Klein AB, Williamson R, Santini MA, Clemmensen C, Ettrup A, Rios M, Knudsen GM, Aznar S (2011). Blood BDNF concentrations reflect brain-tissue BDNF levels across species. Int J Neuropsychopharmacol.

[39] Fung J, Gelaye B, Zhong QY, Rondon MB, Sanchez SE, Barrios YV, Hevner K, Qiu C, Williams MA (2015). Association of decreased serum brain-derived neurotrophic factor (BDNF) concentrations in early pregnancy with antepartum depression. BMC Psychiatry.

[40] Jiang B, Xiong Z, Yang J, Wang W, Wang Y, Hu ZL, Wang F, Chen JG (2012). Antidepressant-like effects of ginsenoside Rg1 are due to activation of the BDNF signalling pathway and neurogenesis in the hippocampus. Br J Pharmacol.

[41] Zhao C, Gammie SC (2014). Glutamate, GABA, and glutamine are synchronously upregulated in the mouse lateral septum during the postpartum period. Brain Res.

[42] Maguire J, Mody I (2008). GABAAR plasticity during pregnancy: relevance to postpartum depression. Neuron.

[43] McEwen AM, Burgess DT, Hanstock CC, Seres P, Khalili P, Newman SC, Baker GB, Mitchell ND, Khudabux-Der J, Allen PS, LeMelledo JM (2012). Increased glutamate levels in the medial prefrontal cortex in patients with postpartum depression. Neuropsychopharmacology.

[44] Xie M, Yan J, He C, Yang L, Tan G, Li C, Hu Z, Wang J (2015). Short-term sleep deprivation impairs spatial working memory and modulates expression levels of ionotropic glutamate receptor subunits in hippocampus. Behav Brain Res.

[45] Treccani G, Gaarn du Jardin K, Wegener G, Muller HK (2016). Differential expression of postsynaptic NMDA and AMPA receptor subunits in the hippocampus and prefrontal cortex of the flinders sensitive line rat model of depression. Synapse.

[46] Schlichter R, Rybalchenko V, Poisbeau P, Verleye M, Gillardin J (2000). Modulation of GABAergic synaptic transmission by the non-benzodiazepine anxiolytic etifoxine. Neuropharmacology.

[47] Guo YY, Liu SB, Cui GB, Ma L, Feng B, Xing JH, Yang Q, Li XQ, Wu YM, Xiong LZ (2012). Acute stress induces down-regulation of large-conductance Ca2+−activated potassium channels in the lateral amygdala. J Physiol.

[48] Mizuki D, Matsumoto K, Tanaka K, Thi Le X, Fujiwara H, Ishikawa T, Higuchi Y (2014). Antidepressant-like effect of Butea Superba in mice exposed to chronic mild stress and its possible mechanism of action. J Ethnopharmacol.

[49] Berry A, Bellisario V, Capoccia S, Tirassa P, Calza A, Alleva E, Cirulli F (2012). Social deprivation stress is a triggering factor for the emergence of anxiety- and depression-like behaviours and leads to reduced brain BDNF levels in C57BL/6J mice. Psychoneuroendocrinology.

[50] Strekalova T, Spanagel R, Bartsch D, Henn FA, Gass P (2004). Stress-induced anhedonia in mice is associated with deficits in forced swimming and exploration. Neuropsychopharmacology.

[51] Cates LN, Roberts AJ, Huitron-Resendiz S, Hedlund PB (2013). Effects of lurasidone in behavioral models of depression. Role of the 5-HT(7) receptor subtype. Neuropharmacology.

[52] Bourin M, Mocaer E, Porsolt R (2004). Antidepressant-like activity of S 20098 (agomelatine) in the forced swimming test in rodents: involvement of melatonin and serotonin receptors. J Psychiatry Neurosci.

